# *In Vitro* Exposure of Leukocytes to HIV Preexposure Prophylaxis Decreases Mitochondrial Function and Alters Gene Expression Profiles

**DOI:** 10.1128/AAC.01755-20

**Published:** 2020-12-16

**Authors:** Emily R. Bowman, Cheryl Cameron, Brian Richardson, Manjusha Kulkarni, Janelle Gabriel, Aaren Kettelhut, Lane Hornsby, Jesse J. Kwiek, Abigail Norris Turner, Carlos Malvestutto, Jose Bazan, Susan L. Koletar, Susanne Doblecki-Lewis, Michael M. Lederman, Mark Cameron, Nichole R. Klatt, Jordan E. Lake, Nicholas T. Funderburg

**Affiliations:** a The Ohio State University, Columbus, Ohio, USA; b Case Western Reserve University, Cleveland, Ohio, USA; c University of Miami, Miami, Florida, USA; d Department of Surgery, University of Minnesota, Minneapolis, Minnesota, USA; e The University of Texas Health Science Center, Houston, Texas, USA

**Keywords:** preexposure prophylaxis, mitochondrial dysfunction, leukocytes, lipids, inflammation, human immunodeficiency virus

## Abstract

The use of antiretroviral therapy (ART) as preexposure prophylaxis (PrEP) is an effective strategy for preventing HIV acquisition. The cellular consequences of PrEP exposure, however, have not been sufficiently explored to determine potential effects on health in individuals without HIV. In this study, peripheral blood mononuclear cells (PBMCs) from people without HIV were exposed to tenofovir disoproxil fumarate (TDF) or emtricitabine (FTC) overnight. Mitochondrial mass and function were measured by flow cytometry and an Agilent XFp analyzer. Monocyte-derived macrophages (MDMs) were differentiated in 20% autologous serum for 5 days in the presence or absence of TDF or FTC, and surface markers, lipid uptake, and efferocytosis were measured by flow cytometry. MDM gene expression was measured using transcriptome sequencing (RNA-seq). Plasma lipids were measured using mass spectrometry. PBMCs exposed to TDF or FTC had decreased maximal oxygen consumption rate (OCR) and reduced mitochondrial mass. Exposure to PrEP also increased reactive oxygen species (ROS) production from monocyte subsets. Compared to MDMs cultured in medium alone, cells differentiated in the presence of TDF (829 genes) or FTC (888 genes) had significant changes in gene expression. Further, PrEP-exposed MDMs had decreased mitochondrial mass and displayed increased lipid uptake and reduced efferocytosis. Plasma biomarkers and lipid levels were also altered *in vivo* in individuals receiving a PrEP regimen. In conclusion, exposure of leukocytes to TDF or FTC resulted in decreased mitochondrial function and altered functional and transcriptional profiles. These findings may have important implications for the metabolic and immunologic consequences of PrEP in populations at risk for HIV acquisition.

## Abstract (precis)

The use of antiretroviral therapy (ART) as preexposure prophylaxis (PrEP) is an effective strategy for preventing HIV acquisition. The cellular consequences of PrEP exposure, however, have not been sufficiently explored to determine potential effects on health in individuals without HIV. In this study, peripheral blood mononuclear cells (PBMCs) from people without HIV were exposed to tenofovir disoproxil fumarate (TDF) or emtricitabine (FTC) overnight. Mitochondrial mass and function were measured by flow cytometry and an Agilent XFp analyzer.

## INTRODUCTION

Eradication of human immunodeficiency virus (HIV) is a global priority, and use of preexposure prophylaxis (PrEP) to limit acquisition of new infections is an important strategy to achieve this goal (1). PrEP is effective at preventing HIV transmission (2), but exposure to antiretroviral therapy (ART) is not without consequences. Few studies, however, have investigated the potential off-target effects of PrEP use in people without HIV. The main FDA-approved form of PrEP is coformulated tenofovir disoproxil fumarate (TDF) and emtricitabine (FTC) (Truvada) taken once daily. Previous work has demonstrated that ART drugs, including nucleoside reverse transcriptase inhibitors (NRTIs), can decrease mitochondrial function (3, 4). Newer NRTIs, including TDF and FTC, are likely less toxic to mitochondria than older drugs (e.g., stavudine and didanosine), but TDF and FTC may also induce mitochondrial dysfunction, potentially through inhibition of mitochondrial DNA (mtDNA) polymerase γ and subsequent decreases in mtDNA content and respiratory chain function (4–6). Mitochondrial dysfunction may result in changes in fatty acid oxidation (FAO) (7) and adipokine levels (8), thereby contributing to adipose tissue dysfunction and circulating lipid profile disturbances and plausibly to increased cardiovascular disease (CVD) risk. Mitochondrial dysfunction may also contribute to local and systemic inflammation through mechanisms related to reactive oxygen species (ROS) production, NF-κB signaling, and inflammasome activation (9). Many of the current studies exploring the effects of ART drugs on cellular mitochondrial function or lipid profiles are performed with people with HIV (PWH), and the inflammatory, immunologic, and metabolic complications associated with the complex pathophysiological disturbances of HIV infection likely complicate the measurement of the direct effects of ART drugs on cellular function.

In this study, we explored the potential functional and phenotypic consequences of *in vitro* exposure of TDF and FTC on peripheral blood mononuclear cells (PBMCs). We also evaluated alterations in lipid profiles and plasma inflammatory biomarkers in a convenience cohort of people initiating PrEP. Our work suggests that exposure of immune cells to PrEP drugs could have unintended adverse effects on cellular function that may contribute to altered innate immune responses and, potentially, cardiometabolic risk in some populations. Although the risk-benefit trade-off using PrEP versus acquisition of HIV undoubtedly favors prevention at the population level, exploring the consequences of PrEP exposure on immune response and metabolism is important to individualizing care.

## RESULTS

### PrEP exposure decreases mitochondrial respiration.

**FIG 1:**
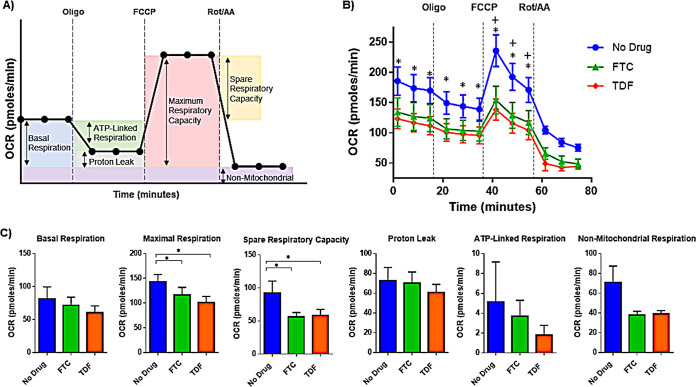
PrEP exposure alters mitochondrial function. (A) Cellular oxygen consumption rate (OCR) kinetics were measured on an Agilent XFp extracellular flux analyzer. As indicated, sequential injections of oligomycin (ATP synthase inhibitor), carbonyl cyanide 4-(trifluoromethoxy) phenylhydrazone (FCCP) (mitochondrial uncoupler), and rotenone/antimycin A (complex I and II inhibitors) were added to the assay media to assess mitochondrial function. (B) PBMCs were exposed to FTC (1 μM) or TDF (1 μM) for 24 h, plated into XFp microplates (500,000 cells/well), and overall OCR kinetics and effects of mitochondrial inhibitors on respiration are shown (+, *P* = 0.05, no drug versus FTC; *, *P* < 0.05, no drug versus TDF; *n* = 6 donors). (C) Basal and maximal respiration, spare respiratory capacity, proton leak, ATP-linked respiration, and nonmitochondrial respiration values were calculated using the Agilent Mito stress test report generator (*, *P* < 0.05).

To assess the effects of PrEP exposure on mitochondrial function, we obtained PBMCs from people without HIV, incubated these cells overnight with FTC or TDF, and measured real-time mitochondrial oxygen consumption rate (OCR) in live cells. Subsequent injections of oligomycin (ATP synthase inhibitor), carbonyl cyanide 4-(trifluoromethoxy) phenylhydrazone (FCCP) (mitochondrial uncoupler), and rotenone/antimycin A (complex I and II inhibitors) were used to assess mitochondrial basal and maximum respiration, spare respiratory capacity, ATP production, proton leak, and nonmitochondrial respiration (Fig. 1A and B) (10, 11). FCCP-induced maximal OCR and spare respiratory capacity, an indicator of a cell’s ability to respond to energetic demand, were significantly decreased in PBMCs exposed to FTC and TDF, indicating mitochondrial dysfunction in PrEP-treated cells (Fig. 1C). We did not detect significant differences in measures of basal respiration, proton leak, or ATP-linked respiration in cells left unstimulated versus cells exposed to PrEP drugs. Although not significantly different among treatment groups, cells exposed to FTC and TDF tended to have reduced nonmitochondrial respiration levels (Fig. 1C). Overall, oxygen consumption rate kinetics were decreased in PrEP-treated PBMCs (Fig. 1B).

**FIG 2:**
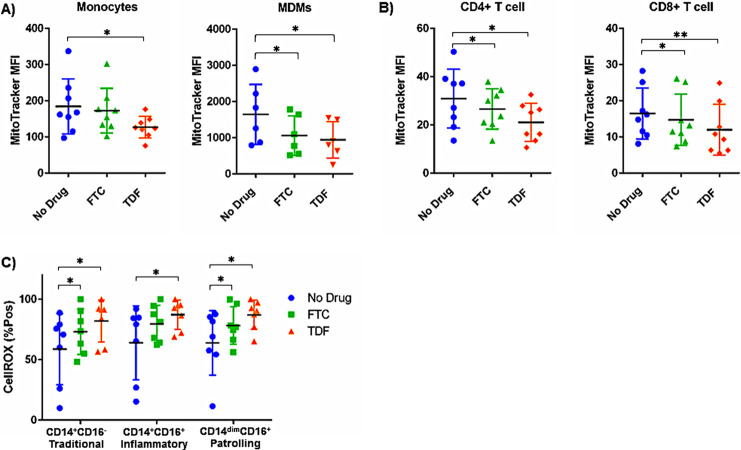
PrEP exposure alters mitochondrial mass measurements in PBMCs. PBMCs (1 × 10^6^/well) were cultured overnight in the presence of FTC (1 μM) or TDF (1 μM). Mitochondrial mass of monocytes and monocyte-derived macrophages (MDMs) (A) and T cells (B) was analyzed by flow cytometry following staining with MitoTracker green (*, *P* < 0.05; **, *P* < 0.01). (C) Intracellular ROS production of ART-exposed monocyte subsets was analyzed by flow cytometry following staining with CellROX Deep Red reagent.

We next asked whether PrEP exposure was sufficient to alter mitochondrial mass, as changes in mitochondrial content may affect cellular respiration (12). PBMCs were stained with MitoTracker green, a green fluorescent stain that localizes to mitochondria in live cells regardless of mitochondrial membrane potential. Monocytes exposed to TDF, and CD4^+^ and CD8^+^ T cells exposed overnight to FTC and TDF had significantly reduced mitochondrial mass, as assessed by MitoTracker green staining (Fig. 2A and B). Monocyte-derived macrophages (MDMs) differentiated for 5 days in the presence of FTC and TDF also had reduced mitochondrial content compared to that of untreated cells (Fig. 2A). Altered mitochondrial function and morphology may lead to increased ROS production, further exacerbating oxidative stress (13). We detected significantly increased levels of intracellular ROS production in monocyte subsets exposed to FTC and TDF in whole blood directly *ex vivo* (Fig. 2C).

### PrEP exposure increases lipid uptake by MDMs.

**FIG 3:**
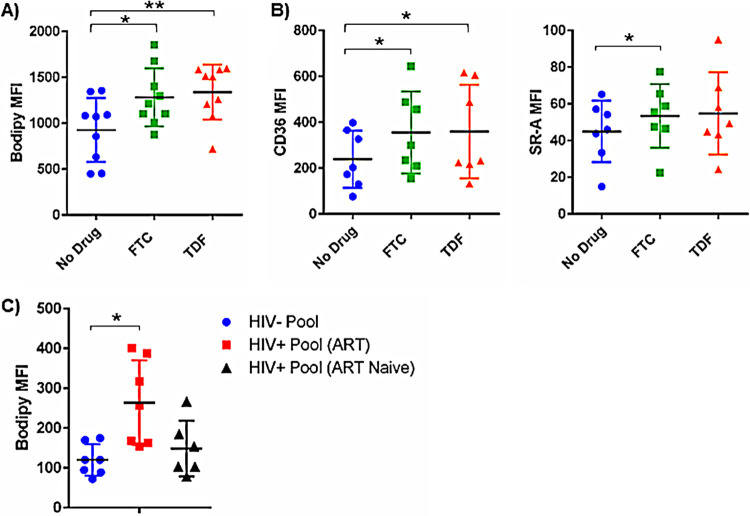
MDMs differentiated in the presence of PrEP display increased lipid accumulation and scavenger receptor expression. (A) Intracellular lipid accumulation was measured by flow cytometry following staining with Bodipy cell-permeative dye. (B) Scavenger receptors CD36 and SR-A were measured by flow cytometry (*, *P* < 0.05; **, *P* < 0.01). (C) PBMCs from people without HIV were differentiated in serum pooled from donors without HIV, donors with HIV on ART (HIV-1 RNA < 40 copies/ml), and ART-naive donors with HIV. MDM lipid accumulation was assessed by flow cytometry analysis of Bodipy staining intensity.

To examine the consequences of PrEP exposure on macrophage phenotype and function, MDMs from participants without HIV were differentiated for 5 days in autologous serum in the presence of FTC and TDF. Intracellular lipid accumulation was increased in MDMs differentiated in the presence of both FTC and TDF (Fig. 3A). Consistent with alterations in lipid uptake, surface expression of lipid-binding scavenger receptors, CD36 and scavenger receptor A (SR-A), was increased on MDMs differentiated in the presence of PrEP drugs.

ART use in PWH has been previously linked to dyslipidemia and altered lipid processing (14); however, the metabolic consequences of using ART for PrEP are unclear. MDMs obtained from people without HIV and differentiated in serum pooled from people without HIV, PWH on ART, or ART-naive PWH displayed different patterns of lipid accumulation. MDMs differentiated in pooled serum from PWH on ART displayed significantly more intracellular lipid accumulation than MDMs differentiated in serum pooled from either people without HIV or ART-naive PWH. These findings may suggest that ART exposure could have adverse metabolic consequences *in vivo* in people with and without HIV.

### Transcriptional profiles are altered in MDMs differentiated in the presence of PrEP.

**FIG 4:**
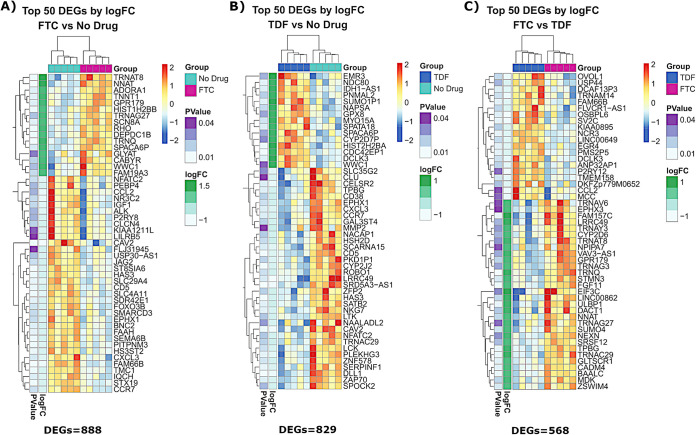
MDMs exposed to PrEP have altered gene expression profiles. Transcript analysis identified differentially expressed genes among MDMs from people without HIV (*n* = 5) differentiated in the presence of medium alone or medium containing FTC (0.1 μM) or TDF (0.1 μM). The top 50 significantly differentially expressed genes (DEGs) are visualized as heat maps, and the data are arranged by *P* value and log fold change (logFC).

**FIG 5:**
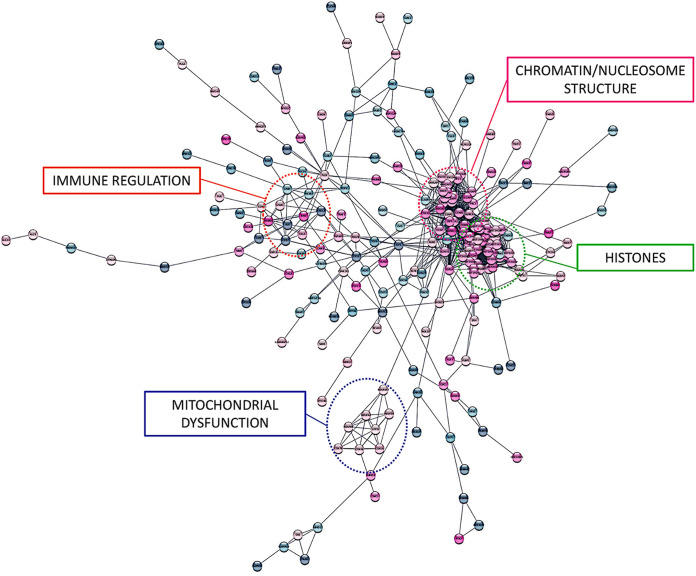
Integrative network analyses of differentially expressed genes (*P* < 0.025) in PrEP-exposed MDMs. Linkages among DEGs are displayed, and nodes demonstrating the most highly interconnected pathways are labeled (Pearson correlation coefficients). Red circles indicate increased gene expression, and blue circles indicated decreased gene expression.

MDM differentiation in the presence of FTC and TDF resulted in distinct transcriptional profiles compared to those of untreated MDMs. We identified 888 differentially expressed genes (DEGs) in FTC-treated MDMs and 829 DEGs in TDF-treated MDMs compared to gene expression in the control group. Additionally, we identified 568 DEGs when comparing FTC and TDF treatment groups, indicating significant divergent effects of each drug on MDM transcript profiles. The top 50 DEGs for each treatment comparison are visualized as heat maps in Fig. 4. PrEP exposure altered numerous signaling pathways associated with mitochondrial dysfunction, inflammatory signaling, and lipid processing (see Fig. S1 in the supplemental material). We identified differential expression of mitochondrial subunit genes (the COX5A/B, COX6A1, NDUFB9, and NDUFA12 genes) and multiple genes involved in inflammation and immune responses (the CCL3, CCR7, CXCL3, CXCR4, STAT2, MYD88, NLRP3, SOCS6, SOD2, ADORA1, and CD48 genes). We also found altered expression of numerous histone genes (e.g., the HIST1H4, HIST2H2A, HIST2H2B, HIST1H3F, and HIST1H1E genes) and genes involved in chromatin/nucleosome structure (e.g., the NDC80, PRC1, CCNF, CCNB1, and NUSAP1 genes). A complete list of significantly altered genes among our treatment groups is provided in Table S1. Network analysis revealed highly interconnected clusters of these histone and chromatin-related genes differentially expressed in MDMs exposed to PrEP (Fig. 5).

**FIG 6:**
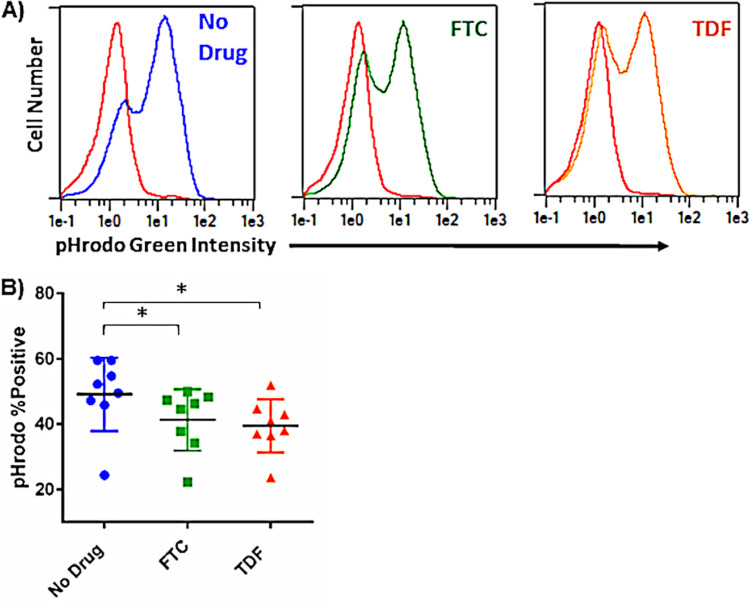
PrEP exposure decreases MDM efferocytosis capacity. MDMs were exposed to pHrodo green-labeled apoptotic Jurkat cells (4:1 ratio of Jurkat cells to MDMs) for 1.5 h at 37°C. Apoptotic cell uptake was analyzed by flow cytometry. (A) Representative histogram overlays; the red line represents MDMs incubated at 0°C under the indicated exposure condition. (B) Summary data reporting percentage of MDMs that have internalized apoptotic cells (*, *P* < 0.05).

MDMs differentiated in the presence of PrEP also displayed reduced expression of the MERTK gene, which encodes a receptor that mediates engulfment and clearance of apoptotic cells (15). Clearance of apoptotic cells, or efferocytosis, by macrophages is important to inhibit inflammation and necrotic core formation in atherosclerotic plaques, and this mechanism is impaired in advanced atherosclerosis (16, 17). Decreased efferocytosis may also underlie inflammation and tissue damage in other sites when apoptotic cells are inefficiently cleared and secondary necrosis occurs (18). To determine whether PrEP exposure altered efferocytosis capacity of MDMs, we incubated MDMs with apoptotic Jurkat cells labeled with a pH-sensitive fluorescent dye to assess phagocytic uptake by MDMs. MDMs differentiated in the presence of FTC and TDF displayed significantly less efferocytosis than control MDMs (Fig. 6).

### Lipids and inflammatory plasma biomarker levels are altered in individuals after initiation of PrEP.

**FIG 7:**
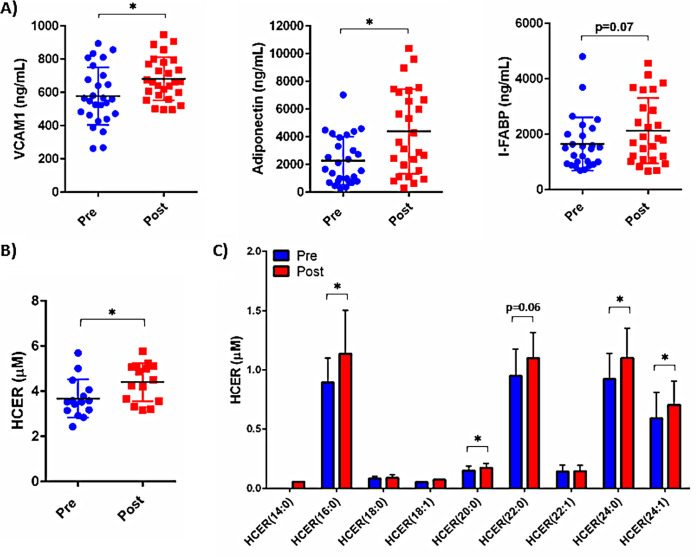
Plasma inflammatory biomarker levels and lipid concentrations are altered following initiation of PrEP. Plasma samples were collected from people without HIV before and after initiation of ART as preexposure prophylaxis (PrEP). (A) Levels of plasma biomarkers, including VCAM-1, adiponectin, and I-FABP, were measured by ELISA pre- and post-PrEP initiation. The average duration of PrEP use among the subjects was 9 months. (B and C) To assess lipid content, plasma samples were analyzed using the direct infusion-tandem mass spectrometry (DI-MS/MS) Lipidyzer platform. Concentrations of total HCER levels (B) and individual HCER species (C) are displayed (*, *P* < 0.05).

To begin exploring *in vivo* consequences of PrEP use, we collected plasma from a convenience sampling of individuals before and after initiation of a PrEP regimen (*n* = 27; median = 7 months of PrEP; mean = 9 months of PrEP; range = 1.7 months to 2.6 years). Several plasma biomarkers were altered in the period following PrEP initiation, including increased soluble vascular cell adhesion molecule 1 (sVCAM-1) and adiponectin levels (Fig. 7A). Additionally, plasma levels of sVCAM-1 were directly associated with adiponectin levels (Fig. S2). We also observed increased levels of intestinal fatty acid binding protein (I-FABP) that approached statistical significance. Further, lipopolysaccharide (LPS)-binding protein (LBP) plasma levels were directly associated with soluble CD14 (sCD14), soluble tumor necrosis factor receptor 1 (sTNFR-1), and sTNFR-2, and zonulin plasma levels were directly associated with oxidized low-density lipoprotein (OxLDL) in individuals after initiation of PrEP (Fig. S2). Duration of PrEP use was directly associated with plasma C-reactive protein (CRP) levels (Fig. S2). *In vitro* exposure of human aortic endothelial cells (HAECs) to FTC and TDF resulted in increased levels of VCAM-1 mRNA, supporting our observation of increased plasma VCAM-1 in PrEP users (Fig. S3).

ART exposure contributes to altered lipid profiles in PWH; however, the consequences of ART exposure on lipidomes of people without HIV have not been adequately explored. Lipidomic analyses of plasma samples from a subset of individuals before and after PrEP exposure (*n* = 15) demonstrated significantly increased concentrations of lipid species PC(18:2/22:5), FFA(22:5), LCER(14:0), and DAG(16:1/22:6) following PrEP use (*P* < 0.05 for all) (data not shown). We also observed increased total concentration of the hexosylceramide (HCER) lipid class (Fig. 7B) and increased levels of the HCER lipid species HCER(16:0), HCER(20:0), HCER(24:0), and HCER(24:1) in individuals taking PrEP (*P* < 0.05 for all) (Fig. 7C).

## DISCUSSION

Much of the current knowledge regarding consequences of ART exposure was gained from studies with PWH. Suppressive ART significantly increases the life spans of PWH, but ART use can also alter metabolic profiles (14, 19) and contribute to CVD risk (20–24). ART initiation is associated with reduced systemic inflammation in PWH, although levels are often not reduced to levels observed in people without HIV (25). Persistent inflammation in PWH may be driven, at least in part, by ART’s adverse effects on mitochondrial function (9). The immunologic consequences of ART use for PrEP in people without HIV have not been well characterized; however, initial studies have reported declines in kidney function and bone mineral density with long-term PrEP use (26–29). A recent study examining ART-related mitochondrial toxicity in HIV-exposed individuals prescribed a postexposure prophylaxis (PEP) regimen demonstrated mitochondrial toxicity after short-term ART use in the absence of HIV infection. Although PEP regimens using newer antiretrovirals, such as TDF plus FTC, showed less mtDNA depletion than regimens that included AZT (30).

Here, we report several metabolic, transcriptional, and functional changes in immune cells following exposure to PrEP drugs. Overnight exposure to TDF or FTC resulted in overall decreased OCR in PBMCs and significantly reduced maximal respiration and spare respiratory capacity. Further, exposure to TDF or FTC also resulted in decreased mitochondrial mass in monocytes and both CD4^+^ and CD8^+^ T cells and increased ROS production from monocyte subsets. These findings demonstrate that exposure to TDF and FTC alters mitochondrial function, potentially as a consequence of decreased mitochondrial mass. Previous work has shown that NRTI-mediated inhibition of DNA polymerase γ leads to depletion of mitochondrial DNA levels and accumulation of mtDNA mutations and, subsequently, mitochondrial dysfunction and altered energy production (31). Previous reports have also demonstrated that NRTIs, such as TDF, FTC, and zidovudine, can alter mitochondrial deoxynucleoside triphosphate (dNTP) pools and impair mitochondrial DNA replication (32), as well as affect mitochondrial function via alterations in cellular metabolism and oxidative stress. Our current report is supportive of these previous findings, and further study is warranted to elucidate mechanisms underlying TDF- and FTC-induced mitochondrial dysfunction observed *in vitro* (33, 34).

We specifically explored the effects of PrEP exposure on MDMs, as these cells have key mitochondrion-dependent effector functions in tissues and are important in the development and progression of CVD. Monocytes migrate from the blood into tissue sites, where they differentiate into macrophages exhibiting a broad phenotypic range, driven by stimuli in the microenvironment (35–39). Here, we demonstrate that MDMs differentiated in the presence of TDF or FTC internalize more lipids and have a decreased capacity to internalize apoptotic cells (efferocytosis). PrEP exposure also increased expression of the innate/lipid receptors CD36 and SR-A, providing one potential mechanism for enhanced lipid uptake. Interestingly, when MDMs from people without HIV were differentiated in the presence of serum pooled from PWH receiving a FTC/TDF/raltegravir regimen, these cells displayed significantly increased intracellular lipid accumulation compared to that of MDMs differentiated in either pooled serum from people without HIV or pooled serum from ART-naive PWH, despite the fact that sera from ART-naive PWH typically contain high levels of inflammatory cytokines, microbial products, and HIV-1 itself (40–42). Our data would suggest the possibility that alterations in lipid uptake induced by factors in serum of PWH were more related to ART exposure than to the circulating proinflammatory cytokines. Further, exposure to TDF or FTC during MDM differentiation resulted in substantial changes in the transcriptional profiles of these cells. Expression of over 800 genes was altered following exposure of MDMs to PrEP. Further, transcriptional network analyses demonstrated interconnected clusters of differentially expressed genes involved in mitochondrial dysfunction, immune regulation, and histone/chromatin modification. We also identified over 500 differentially expressed genes when comparing MDMs differentiated in either TDF or FTC, suggesting that although these drugs are from the same class of ART, some of the mechanisms by which they alter immune cell function are likely different. We did identify 126 genes that were similarly altered among TDF- and FTC-exposed MDMs, including the CCL3, CCR7, COX5B, RUNX2, and STAT2 genes. These *in vitro* results demonstrate that exposure to PrEP drugs can alter the transcriptional, metabolic, and functional capacities of immune cells. Future studies should also examine differential immune response to infection in MDMs exposed to PrEP, as alterations in immune signaling cascades may have important implications for the recognition and response to bacterial and viral ligands.

We also measured *in vivo* changes in lipid levels in individuals who were initiating PrEP. Notably, total HCER levels, as well as concentrations of multiple HCER lipid species, were increased with PrEP use. Ceramides, including HCERs, have been previously linked to cardiometabolic complications and insulin resistance (43–47). Furthermore, HCERs (22:0, 24:0, and 24:1) were previously associated with hepatic inflammation and nonalcoholic steatohepatitis in obese adults (48), and we observed increased concentrations of these HCER species following PrEP initiation. Further studies exploring the changes in the lipidomes of PrEP users and the metabolic complications of ART should be considered.

Plasma biomarkers were also altered with PrEP use. Levels of VCAM-1, adiponectin, and I-FABP were increased in plasma samples following PrEP exposure. *In vitro* exposure of primary human aortic endothelial cells to FTC and TDF similarly resulted in increased expression of VCAM-1. Increased circulating concentrations of endothelial activation markers, such as VCAM-1, have been described for PWH and are linked to chronic immune activation. I-FABP levels are indicative of gut epithelial barrier integrity and are linked to mortality in PWH (49, 50), and gastrointestinal side effects associated with PrEP initiation have been described (51). Unique characteristics, specific to individual PrEP users in this cohort, including lifestyle factors, underlying comorbidities, and/or coinfections, may influence levels of inflammatory markers and their changes over time, and we did not have access to detailed medical record information from this cohort. The need for well-designed trials exploring the immunologic and metabolic consequences of ART use in people without HIV is underscored by our results and the recent findings by Korencak et al., which demonstrated that exposure of immune cells to different classes of ART drugs, both *in vitro* and *in vivo*, resulted in changes in metabolic and functional signatures from these cells (34). Importantly, exposure to integrase inhibitors (dolutegravir and elvitegravir) resulted in decreased polyfunctionality of CD4^+^ T cells and instead promoted stress responses (increased TNF-α and ROS). In ART-treated women with HIV, monocyte mitochondrial and glycolytic dysfunction was associated with body composition (33), suggesting that future studies with PrEP users should also explore changes in body composition and mitochondrial function as well.

Exposure of immune cells to ART, as HIV-1 treatment or as PrEP, likely has important consequences on the metabolic and functional capacities of innate and adaptive immune cells and may affect immune responses and cardiometabolic risk in both populations. The risk of taking PrEP versus the benefit of avoiding HIV acquisition clearly favors prevention, regardless of the off-target effects of ART exposure on immune cell function; yet these findings merit further study to optimize PrEP methods. New PrEP formulations, including FTC and tenofovir alefenamide (Descovy) and the long-acting injectable PrEP cabotegravir are being administered for HIV prevention; the effects of these drugs on immune cells, the lipidome, and mitochondrial function should be explored (52). Further studies are needed to adequately understand the *in vivo* consequences of long-term PrEP exposure on immune cell mitochondrial function and gene expression, lipid metabolism, and risk for adverse health outcomes that may alter PrEP risk/benefit considerations.

## MATERIALS AND METHODS

### Sample collection.

Blood samples were collected in EDTA-containing Vacutainer tubes (BD Biosciences) for PBMC isolation and whole-blood stimulations. For serum collection, blood was drawn into serum separation tubes (SST; BD Biosciences) and centrifuged for 15 min at 800 × *g*. Study participants initiating PrEP (*n* = 27) were enrolled at Case Western University following written informed consent. Longitudinal plasma samples were collected before and after initiation of PrEP (median = 7 months of PrEP; mean = 9 months of PrEP; range = 1.7 months to 2.6 years).

### PBMC isolation and cell culture.

PBMCs were freshly isolated from donors without HIV by centrifugation over Ficoll-Hypaque and cultured in RPMI 1640 supplemented with 10% autologous serum. For differentiation of monocyte-derived macrophages (MDMs), donor PBMCs (2 × 10^6^/ml) were cultured in Teflon wells (Savillex) for 5 days in RPMI 1640 supplemented with 20% autologous serum. On day 5, cells were removed from Teflon and MDMs were purified by adherence to plastic for 3 h. For MDM pooled-serum experiments, PBMCs were isolated from donors without HIV (*n* = 7) and differentiated in Teflon wells for 5 days in 20% serum pooled from donors without HIV, donors with HIV on ART (HIV-1 RNA < 40 copies/ml), or ART-naive donors with HIV. Human aortic endothelial cells (HAECs) were purchased from PromoCell and grown in low-serum endothelial cell growth medium with supplement mix (PromoCell).

Lyophilized FTC and TDF stocks were obtained from the NIH AIDS Reagent Program and solubilized in water. For *in vitro* PrEP experiments, PBMCs (1 × 10^6^/well) were exposed to FTC (1 μM) and TDF (1 μM) for 24 h, and MDMs were differentiated for 5 days in the presence of FTC (0.1 μM) and TDF (0.1 μM). MDM morphology had a healthier “fried egg” appearance at the lower concentration of 0.1 μM for the longer period (5 days) than at the higher concentration of 1 μM for the shorter period (24 h) for PBMC experiments. Clinically relevant concentrations were selected based on initial dose response assays (53–55). Cells maintained ∼100% viability until >1,000 μM drug exposure (24 h).

### Flow cytometry.

Cells harvested from Teflon were washed and blocked in 10% human AB serum (Sigma) for 60 min. MDMs were then stained for 30 min in the dark on ice, washed, and fixed in 1% paraformaldehyde. Monocytes and MDMs were identified by granularity, size, and surface expression of CD14 and CD16 (anti-CD14 Pacific blue and anti-CD16 phycoerythrin [PE]; BD Pharmingen). MDM scavenger receptor expression was measured using anti-CD36 (allophycocyanin [APC]) (BD Pharmingen) and anti-SR-A (CD204, fluorescein isothiocyanate [FITC]) (Miltenyi Biotec). T cells were identified by granularity, size, and surface expression of CD3 (APC), CD4 (Pacific blue), and CD8 (peridinin chlorophyll protein [PerCP]) (BD Pharmingen for all). For analysis of mitochondrial mass, cells were stained with MitoTracker green (200 nM; Thermo Fisher) for 30 min at 37°C. To evaluate intracellular lipid accumulation, MDMs were stained with 0.25 μg/ml of Bodipy 493/503 (Life Technologies) for 20 min in the dark at room temperature and then analyzed by flow cytometry. Cells were analyzed using a Miltenyi MACSQuant Analyzer 10 flow cytometer and MACSQuant analysis software. Statistical analysis was performed in GraphPad Prism 6, and paired *t* tests were used to compare flow cytometric data obtained from cells exposed to TDF or FTC and no-drug controls.

### Measurement of intracellular reactive oxygen species.

CellROX Deep Red (Thermo Fisher) is a cell-permeative probe that exhibits excitation/emission maxima at ∼640/665 upon oxidation by reactive oxygen species (ROS). To measure ROS, whole blood from donors without HIV was exposed to FTC (1 μM) or TDF (1 μM) for 3 h and then incubated with 5 μM CellROX Deep Red at 37°C for 30 min. Blood samples were then incubated for 15 min on ice with FACS lysis buffer (BD Biosciences) and washed with flow cytometry wash buffer in preparation for flow cytometry analysis.

### Soluble markers.

Levels of the following inflammatory plasma biomarkers were measured by enzyme-linked immunosorbent assay (ELISA; R&D Systems unless stated otherwise): soluble CD14 (sCD14), tumor necrosis factor receptor 1 (TNFR-1), TNFR-2, vascular cell adhesion molecule 1 (VCAM-1), C-reactive protein (CRP), LPS-binding protein (LBP), intestinal fatty acid binding protein (I-FABP), zonulin (Promokine), and oxidized low-density lipoprotein (OxLDL; Mercodia). Paired *t* tests were used to compare biomarker levels before and after PrEP initiation. Spearman correlations are reported for relationships among plasma biomarker levels.

### Metabolic analyses.

Mitochondrial oxygen consumption rate (OCR) kinetics were measured by an Agilent XFp analyzer (*n* = 7) using the Mito stress test kit (Agilent Technologies) as per the manufacturer’s protocol. Prior to the assay, Agilent XFp cell culture miniplates were coated with Cell-Tak (22.4 μg/ml; Corning). PBMCs were exposed overnight to FTC (1 μM) or TDF (1 μM), centrifuged and resuspended in Agilent XF assay medium, and plated (500,000 cells/well) into Cell-Tak-coated miniplates to adhere PBMCs for analysis by the Agilent XFp analyzer. In this assay, subsequent injections of oligomycin (ATP synthase inhibitor), carbonyl cyanide 4-(trifluoromethoxy) phenylhydrazone (FCCP) (mitochondrial uncoupler), and rotenone/antimycin A (complex I and II inhibitors) are added to the assay media to assess mitochondrial function. Optimal cell density and FCCP concentration (1 μM) were selected based on initial titration and dose-response assays. Statistical and data analysis was performed using the Seahorse XF Cell Mito stress test report generator and supported Wave software.

### Efferocytosis assays.

MDM efferocytosis activity was measured using apoptotic Jurkat cells labeled with pHrodo green (Thermo Fisher), a pH-sensitive dye that fluoresces only in the acidic environment of phagosomes. Jurkat cells were incubated with dexamethasone (100 μM) overnight, and induction of apoptosis was verified using the Dead Cell Apoptosis kit (Thermo Fisher). Apoptotic Jurkat cells were washed with PBS and labeled with 20 ng/ml of pHrodo green (Thermo Fisher) for 30 min at room temperature. Labeled apoptotic cells were resuspended in culture medium and used immediately.

After 5 days of differentiation in the presence of FTC (0.1 μM) or TDF (0.1 μM), MDMs were plated in 12-well culture plates (3 × 10^5^ MDMs/ml) and purified by adherence to plastic for 3 h. MDMs were resupplemented with PrEP drugs and incubated overnight. pHrodo green-labeled apoptotic Jurkat cells were added to MDMs (4:1 ratio of Jurkat cells to MDMs) and incubated at 37C for 1.5 h. Uptake of apoptotic cells was analyzed by flow cytometry and quantified by mean fluorescence intensity (MFI). For negative controls and background correction, MDMs were incubated on ice for 1.5 h with pHrodo green-labeled apoptotic Jurkat cells. Final reported fluorescence intensity was calculated by subtracting the intensity of cells incubated at 0°C from that of cells incubated at 37°C. Paired *t* tests were used for statistical comparisons between the no-drug control and FTC- or TDF-exposed cells.

### RNA isolation and transcriptomic analyses.

Total MDM RNA was isolated using the RNeasy RNA isolation kit (Qiagen). Transcriptome sequencing (RNA-seq) libraries were prepared with standard TruSeq stranded total RNA (Ribo-Zero) kits (Illumina) or Clontech Smart-Seq Ultra Low kits plus Nextera XT adaptors (Clontech) and sequenced using an Illumina HiSeq2500 instrument (2 × 125 cycles, 6-plex, ~30 million paired reads/sample), i.e., beyond the depth plateau necessary to fully characterize the transcriptome and capture information encoded by rarer alternative transcripts and isoforms. Our Bioconductor “R” pipeline is data type agnostic and was used to demultiplex, quality control (QC), align (UCSC Hg38 human reference), annotate, and count the transcripts. Differential expression analysis was performed using EdgeR and gene-by-gene contrasts (*t* or F test) between groups. A false-discovery rate (FDR) of 5% was employed to correct for multiple testing. Pathway analyses were performed using gene set variation analysis (GSVA), gene sets from MSigDB, and Ingenuity Pathway analysis.

For HAEC quantitative PCR (qPCR) analysis, the iScript cDNA synthesis kit and iQ SYBR green Supermix were used (Bio-Rad). Paired *t* tests were used for statistical calculations. VCAM-1 transcript levels were analyzed using the following primer set: forward, TTTGACAGGCTGGAGATAGACT, and reverse, TCAATGTGTAATTTAGCTCGGCA.

### Lipid measurement.

Plasma lipids were analyzed using the direct infusion-tandem mass spectrometry (DI-MS/MS) Lipidyzer platform (Sciex, MA), which identifies and quantifies ∼1,100 biological lipids covering 13 lipid classes (e.g., free fatty acids, ceramides, hexosylceramides, diacylglycerols, and triacylglycerols). The Lipidyzer platform methodology has been described in detail elsewhere (56), but briefly, lipids were extracted from 100 μl of plasma using a modified Bligh-Dyer method. Over 50 stable isotope-labeled internal standards spanning all 13 lipid classes were added to each sample prior to extraction for accurate quantitation. Extracts were reconstituted in dichloromethane/methanol (1:1) and analyzed using DI-MS/MS with differential mobility spectrometry (DMS) separation. A Shimadzu liquid chromatography (LC) system was used for automated infusion of each plasma extract and for pumping running and rinse solutions through the lines. Plasma extracts were infused into a 5500 QTRAP MS/MS with SelexION DMS technology (Sciex) and lipid species were targeted and quantitated using optimized MS/MS transitions. Data were generated using the Lipidomics workflow manager software (Sciex). Data are reported as concentrations (micromolar) and relative fatty acid composition (moles percent) of total lipid classes and individual lipid species. Paired *t* tests were used for comparison of plasma lipid concentrations in individuals before and after PrEP initiation. *P* values of <0.05 were considered statistically significant.

## Supplementary Material

Supplemental file 1

Supplemental file 2
