# Supplementary material for: In Vitro Exposure of Leukocytes to HIV Preexposure Prophylaxis Decreases Mitochondrial Function and Alters Gene Expression Profiles
Source: Antimicrob Agents Chemother. 2020 Dec 16;65(1):e01755-20. doi: 10.1128/AAC.01755-20 (PMC7927818; doi:10.1128/AAC.01755-20)
Supplement: Supplemental file 1 [file AAC.01755-20-s0001.pdf]

## Supplemental Figures

### Supplemental Figure 1.

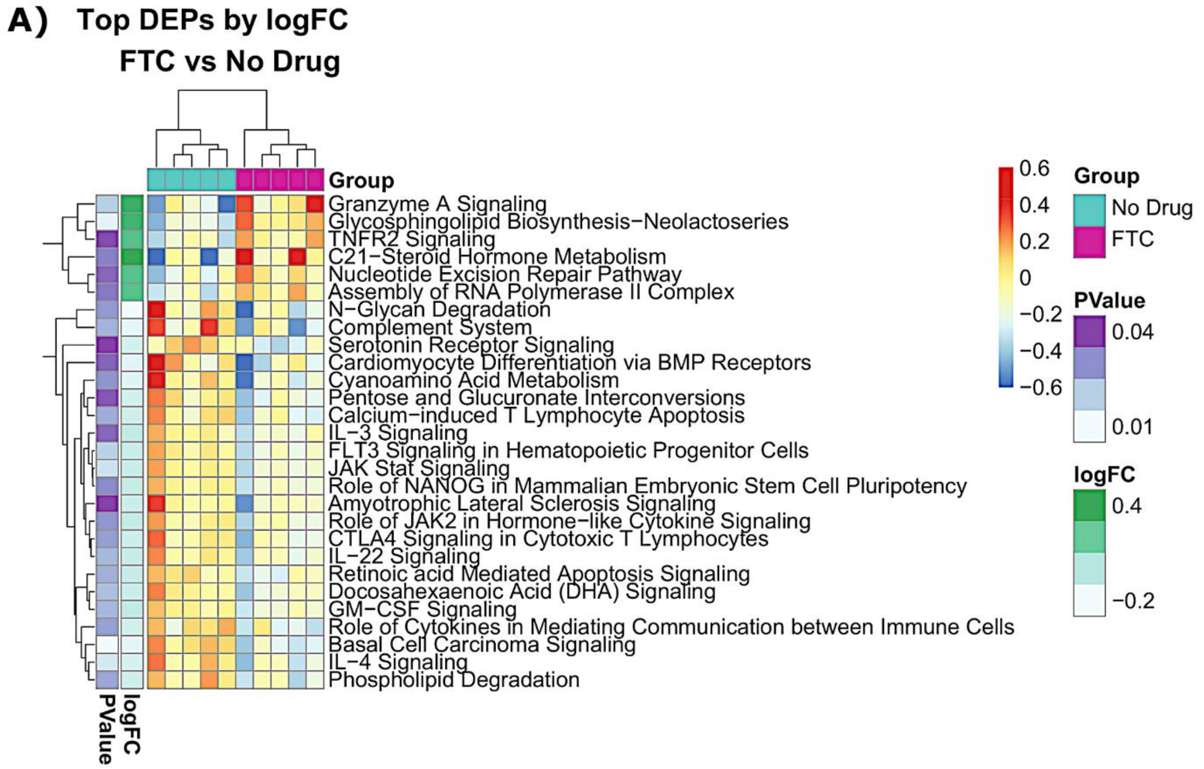


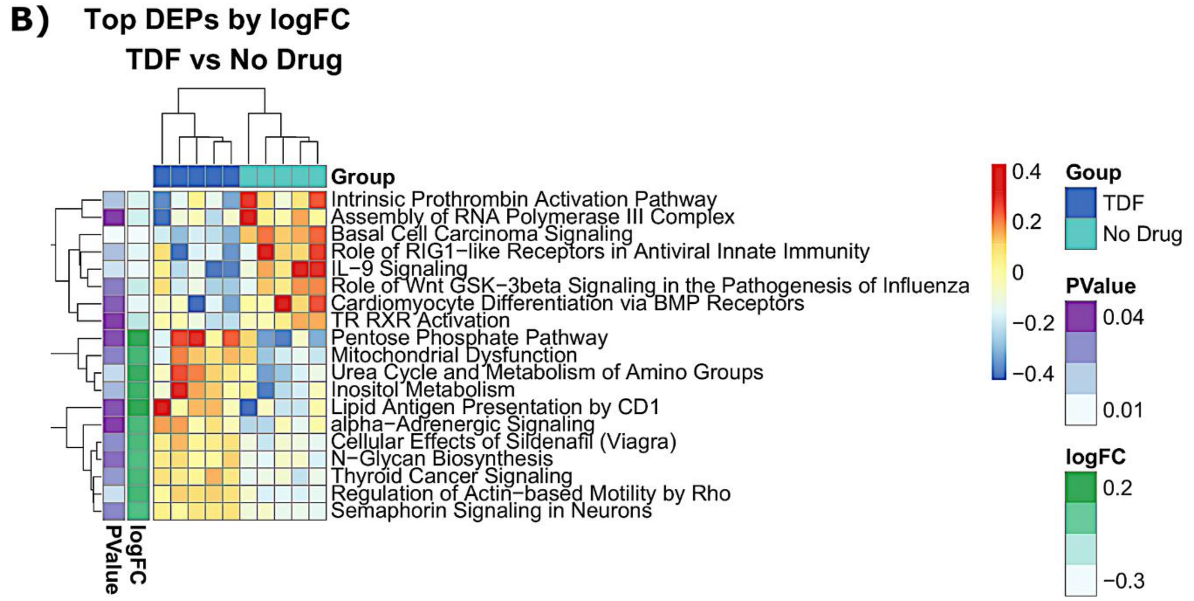

**Supplemental Figure 1.** Exposure to PrEP drugs altered MDM signaling pathway activation. Differentially expressed pathways (DEPs) were identified using gene set variation analysis (GSVA). Data are arranged by p-value and log fold change (LogFC).

**Supplemental Figure 2.**

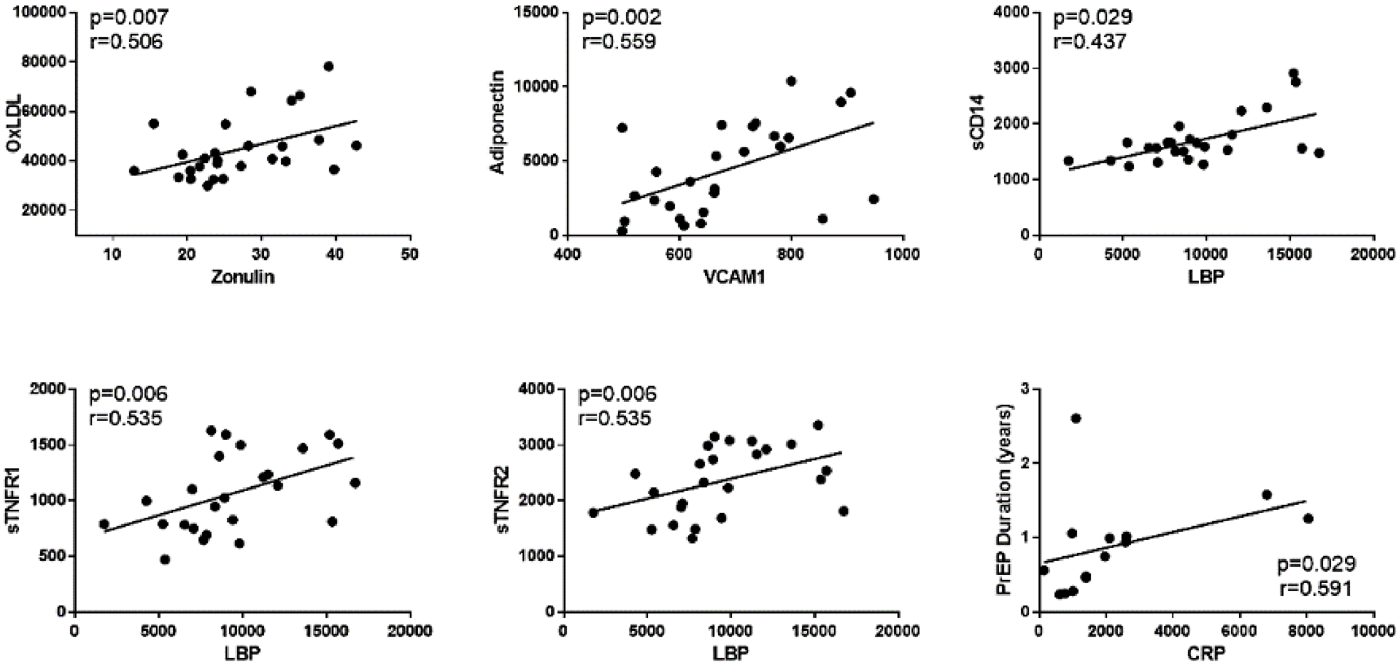


**Supplemental Figure 2.** Spearman correlations are reported for relationships among plasma inflammatory biomarkers.

**Supplemental Figure 3.**

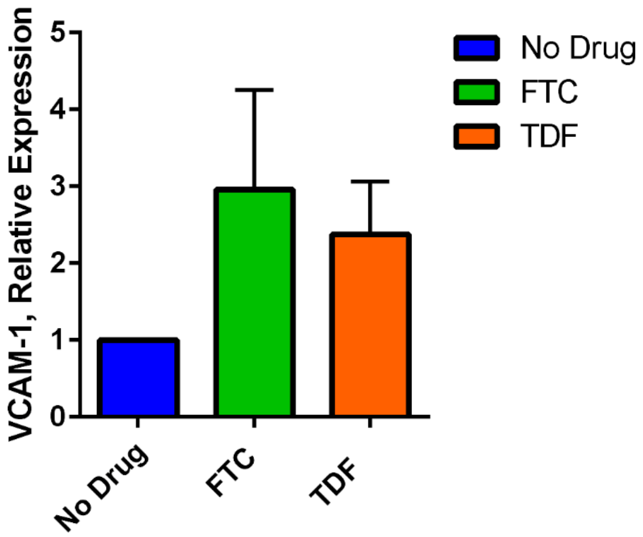


**Supplemental Figure 3**. Primary human aortic endothelial cells were exposed to FTC (1 mM) or TDF (1 mM) for 24h, and were subjected to quantitative PCR analysis. Fold change expression levels of VCAM-1 are displayed relative to no drug treatment controls. (*$p<0.05$)
